# Transcription factor HBP1 is a direct anti-cancer target of transcription factor FOXO1 in invasive oral cancer

**DOI:** 10.18632/oncotarget.14653

**Published:** 2017-01-14

**Authors:** Chien-Yi Chan, Shih-Yi Huang, Jim Jinn-Chyuan Sheu, Mendel M. Roth, I-Tai Chou, Chia-Hsien Lien, Ming-Fen Lee, Chun-Yin Huang

**Affiliations:** ^1^ Department of Nutrition, China Medical University, Taichung, Taiwan; ^2^ School of Nutrition and Health Sciences, Taipei Medical University, Taipei, Taiwan; ^3^ Institute of Biomedical Sciences, National Sun Yatsen University, Kaohsiung, Taiwan; ^4^ Department of Health and Nutrition Biotechnology, Asia University, Taichung, Taiwan; ^5^ GB Lifesciences, San Diego, CA, USA; ^6^ Department of Nutrition and Health Sciences, Chang Jung Christian University, Tainan, Taiwan

**Keywords:** FOXO1, HBP1, oral cancer

## Abstract

Either FOXO1 or HBP1 transcription factor is a downstream effector of the PI3K/Akt pathway and associated with tumorigenesis. However, the relationship between FOXO1 and HBP1 in oral cancer remains unclear. Analysis of 30 oral tumor specimens revealed that mean mRNA levels of both FOXO1 and HBP1 in non-invasive and invasive oral tumors were found to be significantly lower than that of the control tissues, and the status of low FOXO1 and HBP1 (< 0.3 fold of the control) was associated with invasiveness of oral tumors. To investigate if HBP1 is a direct transcription target of FOXO1, we searched potential FOXO1 binding sites in the *HBP1* promoter using the MAPPER Search Engine, and two putative FOXO1 binding sites located in the *HBP1* promoter –132 to –125 bp and –343 to –336 bp were predicted. These binding sites were then confirmed by both reporter gene assays and the *in cellulo* ChIP assay. In addition, Akt activity manipulated by PI3K inhibitor LY294002 or Akt mutants was shown to negatively affect FOXO1-mediated *HBP1* promoter activation and gene expression. Last, the biological significance of the FOXO1-HBP1 axis in oral cancer malignancy was evaluated in cell growth, colony formation, and invasiveness. The results indicated that HBP1 knockdown potently promoted malignant phenotypes of oral cancer and the suppressive effect of FOXO1 on cell growth, colony formation, and invasion was alleviated upon HBP1 knockdown in invasive oral cancer cells. Taken together, our data provide evidence for HBP1 as a direct downstream target of FOXO1 in oral cancer malignancy.

## INTRODUCTION

The forkhead box O (FOXO) transcription factor family shares a conserved ‘forkhead box’ DNA-binding domain and is consisted of four members in mammals: FOXO1, FOXO3a, FOXO4 and FOXO6 [1]. FOXO factors are involved in a wide range of biological processes, including cell cycle arrest, apoptosis, DNA repair, glucose metabolism, oxidative stress resistance and longevity [1]. The biological activity of FOXO factors is mostly dependent on the post-translational modification of phosphorylation, acetylation, or ubiquitination, thereby determining their intracellular trafficking [2]. Among FOXO factors, increased p-FOXO1 or decreased FOXO1 expression is often associated with tumorigenesis [3]. FOXO1 may exert its tumor suppression function through its transcription-dependent expression of growth arrest and apoptotic-related genes, including *p15*, *p19*, *NOXA*, *FasL*, *TRAIL*, and *Bim* [4–6]. The promoters of these genes comprise the FOXO-recognized element with the consensus sequence T/C-G/A-A-A-A-C-A-A [7]. Together, FOXO1 appears to be a potential therapeutic target of anticancer reagents.

HMG box-containing protein 1 (HBP1) is a regulator of cell cycle exit and cell differentiation [8]. Overexpression of HBP1 leads to an inhibition of the G1-S phase transition [9]. Loss-of-function HBP1 variants have been isolated in myeloid leukemia and breast cancers [10, 11]. Conversely, ectopic or chemical-induced expression of HBP1 results in growth arrest, apoptosis, or differentiation in cancer cell lines, including oral cancer [12–15]. Together, these findings suggest a role of HBP1 in tumor suppression. HBP1 exerts its repression function through an HMG box DNA-binding domain and an AXH transcriptional repression domain. Genes down-regulated by HBP1 include *p47phox*, *N-myc*, *c-myc*, *CCND1*, and *MIF* [9, 14, 16, 17].

Studies of HBP1 have been focused on the finding of its transcription downstream targets. How the *HBP1* promoter is regulated remains unclear. In a previous study, we demonstrated that HBP1 is a downstream effector of the EGFR (epidermal growth factor receptor))/Akt pathway in oral cancer [15]. Subsequently, a recent report illustrated that HBP1 is direct target of growth factors-mediated PI3K/Akt/FOXO pathway in various types of cancer [18]; however, whether or not FOXO1 regulates HBP1 expression in oral cancer remains unclear. In this study, we reported that both HBP1 and FOXO1 were down-regulated in a subset of oral tumor specimen. Ectopic expression of FOXO1 led to increased *HBP1* promoter activity and HBP1 expression in oral cancer cells. Indeed, FOXO1-influencing factors, such as Akt activity, also accordingly affect *HBP1* promoter activity and HBP1 expression. The direct binding of FOXO1 onto the *HBP1* promoter was evidenced by the identification of two putative FOXO1 consensus sites in its *cis-acting* region. Furthermore, the biological significance of FOXO1-mediated HBP1 expression was demonstrated by the experiments of colony formation and cell invasion in oral cancer cells. Together, the current study provided evidence that FOXO1 and HBP1 function coordinately as tumor suppressors in invasive oral cancer.

## RESULTS

### Low FOXO1/low HBP1 expression predicts invasiveness of oral cancer

FOXOs transcription activity is tightly regulated by the Akt signaling [19]. Previously, we demonstrated that HBP1 functions as a downstream effector of the EGFR/Akt signaling pathway in oral cancer [15]. Both FOXOs and HBP1 are negatively regulated by Akt. FOXOs are directly modified by Akt phosphorylation for subsequent degradation; however, how HBP1 expression is down-regulated by Akt remains unclear in oral cancer. Using the MAPPER Search Engine to predict the putative transcription factor binding sites (TFBSs) in the *HBP1* promoter, we identified several potential FOXO1 binding sites in the proximal region of the *HBP1* promoter. Therefore, we hypothesized that FOXOs may play a crucial role in Akt-mediated suppression of HBP1 expression. Hence, the current study is aimed at investigating the putative transcriptional regulation of HBP1 by FOXO1 and, collectively, the biological significance of FOXO1 and HBP1 in oral cancer.

**Figure 1:**
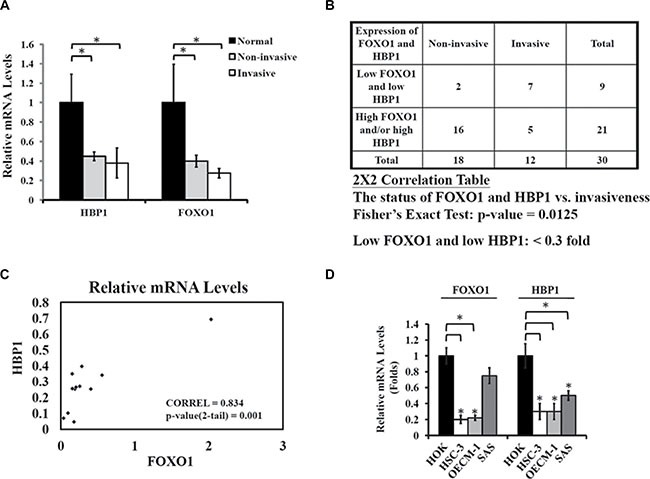
Coordinate down-regulation of FOXO1 and HBP1 in oral cancer (**A**) Quantitative analysis of FOXO1 and HBP1 expression levels in oral tumor specimens. RT-qPCR was used to measure FOXO1 and HBP1 mRNA levels of 30 oral tumor specimens. All 30 tumor specimens were divided into two groups, non-invasive (*n* = 18, pN *=* 0, no metastasis) and invasive (*n* = 12, pN > 0, lymph node metastasis). The mean mRNA levels of FOXO1 and HBP1 from 9 adjacent normal tissue specimens were set as 1 with GAPDH as an internal control. The mean mRNA levels of FOXO1 and HBP1 from non-invasive and invasive oral tumor specimens were significantly lower than that of control, normal tissues. Values were expressed as mean ± S.E.M. (*p < 0.05). (**B**) Association of the FOXO1/HBP1 expression status and invasiveness. Tumors with low FOXO1 and HBP1 (< 0.3-fold of normal) expression are associated with invasiveness in oral cancer specimens. The 2 × 2 correlation table and Fisher's exact test were used, with a significant two-sided value, *p* = 0.0125. (**C**) Correlation between FOXO1 and HBP1 expression in invasive oral tumor specimens. The relative mRNA levels of FOXO1 and HBP1 from 12 invasive oral specimens were under Pearson correlation examination with a coefficient of correlation 0.835 (*p* = 0.001). (**D**) The mRNA levels of FOXO1 and HBP1 in oral cancer cell lines. Total RNA were extracted from similar cell density of HOK human oral keratinocytes, and HSC-3, OECM-1, and SAS oral cancer cell lines and subjected to RT-qPCR analysis for both FOXO1 and HBP1 expression. The mRNA levels of FOXO1 and HBP1 were expressed as relative to 18S, an internal reference (*p < 0.05 as compared with HOK).

First, we examined the association of FOXO1 and HBP1 with oral tumorigenicity by comparing the mRNA levels of FOXO1 and HBP1 from 30 oral tumor specimens obtained from China Medical University Hospital (Taichung, Taiwan) with those of nine normal tissues. The mean mRNA level of either HBP1 or FOXO1 in non-invasive and invasive oral tumors (lymph node metastasis) was significantly lower than those of control normal tissues (Figure 1A). Reduced expression of both FOXO1 and HBP1 was also found in two tongue squamous cell carcinoma datasets in the Oncomine database (Supplementary Figure 1). Further analysis indicated a correlation of a low-HBP1 and FOXO1 status (< 0.3 fold) with the aggressiveness of oral tumors (Figure 1B). Indeed, when we tested the correlation between HBP1 and FOXO1 expression in aggressive oral cancer specimens, we found that HBP1 mRNA levels were positively correlated with FOXO1 mRNA levels in invasive oral cancer (Figure 1C). Furthermore, both FOXO1 and HBP1 mRNA levels were significantly lower in three EGFR over-expressing oral cancer cells tested than the normal human oral keratinocyte (HOK) (Figure 1D). These clinical data suggest that a combination of low HBP1 and low FOXO1 status might determine oral cancer malignancy, and that HBP1 and FOXO1 expression might be coordinately regulated.

### FOXO1 transcriptionally induces HBP1 expression

**Figure 2:**
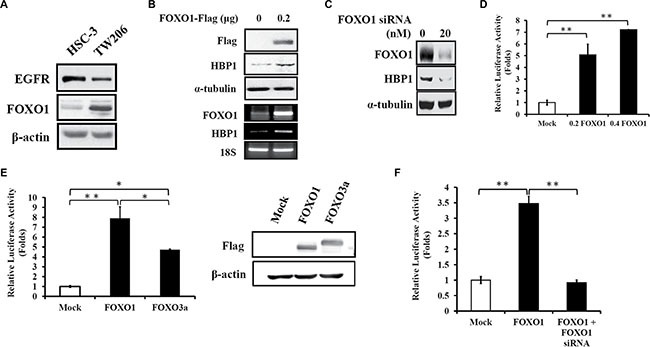
FOXO1 induces *HBP1* gene expression in oral cancer (**A**) The protein levels of EGFR and FOXO1 in HSC-3 and TW206 oral cancer cell lines. Cell lysates from these two cell lines were isolated and then subjected to Western blotting analysis for the detection of endogenous EGFR and FOXO1. (**B**) HSC-3 cells were transfected with pcDNA3-FOXO1-Flag (0.2 μg) or empty vector pcDNA3 for 24 h, and then cell lysates were collected for analysis of the protein levels of FOXO1-Flag, HBP1 and α-tubulin by Western blotting (upper panel). Total RNA extracted from 24 h of FOXO1-Flag-transfected HSC-3 cell was subjected to quantification of FOXO1 and HBP1 mRNA expression by RT-PCR with 18S as internal control (lower panel). (**C**) Expression levels of FOXO1, HBP1, and α-tubulin were examined by Western blotting in TW206 oral cancer cells with FOXO1-specific siRNA transfection for 24 h. (**D**–**F**) Effect of FOXO1 on *HBP1* promoter. HEK-293T cells seeded in a 24-well plate were transfected with (D) increasing amount of FOXO1 (0, 0.2, 0.4 μg), (E) FOXO1 or FOXO3a (0.4 μg) along with a 2-kb *HBP1* promoter-luciferase construct (0.4 μg) for 24 h. (E, upper panel) Luciferase intensities were measured and normalized to β-galactosidase activities (*p < 0.05; **p < 0.01), and (E, lower panel) expression levels of transfected Flag-FOXO1 and Flag-FOXO3a cDNA were detected using anti-Flag antibody by Western blotting analysis. (F) HSC-3 cells in a 24-well plate were transfected with FOXO1 (0.2 μg) and FOXO1 siRNA (20 nM) together with a 2-kb *HBP1* promoter-luciferase construct (0.4 μg) for 24 h. Luciferase intensities were measured and normalized to β-galactosidase activities (**p < 0.01).

To illustrate FOXO1 regulation of HBP1 expression in oral cancer, first, we examined if HBP1 expression levels are in concert with FOXO1 levels. Ectopic expression of FOXO1 in HSC-3 cells with low endogenous FOXO1 levels (Figure 2A) resulted in increased expression of HBP1 protein and mRNA (Figure 2B). Similarly, knockdown of FOXO1 expression with FOXO1-specific siRNA led to reduced HBP1 expression in TW206 oral cancer cells with high level of endogenous FOXO1 (Figure 2C). These data suggest that HBP1 might be transcriptionally regulated by FOXO1. Indeed, ectopic expression of FOXO1 cDNA (0.2 and 0.4 μg) in 293T cells for 24 h enhanced the transcriptional activity of a 2-kb *HBP1* promoter by 5 and 7 folds, respectively (Figure 2D). In addition, FOXO1 exhibited stronger activation of the 2-kb *HBP1* promoter than FOXO3a (Figure 2E) although FOXO1 and FOXO3a share highly similar sequence [20]. FOXO1-induced activation of the 2-kb *HBP1* promoter was also observed in HSC-3 oral cancer cells, and the induction was abolished when co-transfected with FOXO1-specific siRNA (Figure 2F). Taken together, these results indicate that FOXO1 is able to induce HBP1 expression through transcription.

### FOXO1 activity is essential for the FOXO1-mediated HBP1 expression

**Figure 3:**
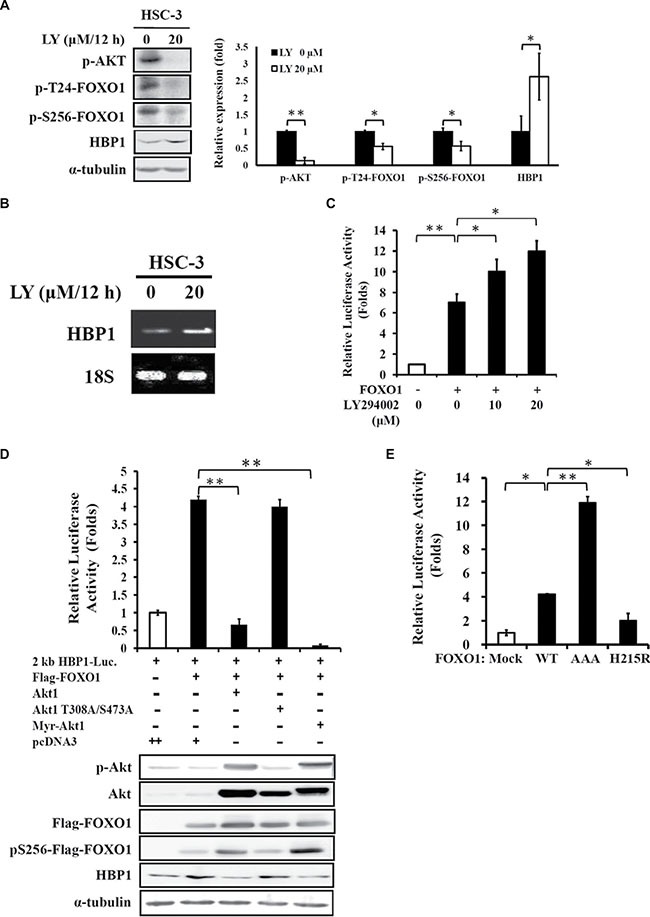
HBP1 expression is regulated by FOXO1 activity (**A**–**C**) Effect of LY294002 on FOXO1-mediated HBP1 expression. (A) HSC-3 cells were treated with LY294002 (20 μM) up to 12 h, followed by detection of p-Akt, p-FOXO1, HBP1, and α-tubulin expression by Western blotting analysis. One representative experiment out of three independent experiments is shown. Values are mean ± SD (*p < 0.05 and **p < 0.01 as compared with vehicle alone). (B) Total RNA isolated from LY294002-treated HSC-3 cells was subjected to RT-PCR analysis for the measurement of HBP1 mRNA level with 18S as an internal control. (**C**) HEK-293T cells were transfected with a 2-kb *HBP1* promoter-luciferase construct, FOXO1 expressing vector, and β-galactosidase plasmid for 24 h, followed by treatment of increasing concentration of LY294002 (0–20 μM) for additional 24 h. Luciferase intensities were measured and normalized to β-galactosidase activities (*p < 0.05; **p < 0.01). (**D**) Effect of Akt activity on FOXO1-mediated HBP1 expression. A 2-kb *HBP1* promoter-luciferase construct (0.2 μg), a β-galactosidase plasmid (0.1 μg), and FOXO1-Flag cDNA (0.2 μg) were co-transfected with 0.2 μg of pcDNA3 empty vector, Akt1, Akt1 T308A/S473A mutant, or constitutively active Myr-Akt1 vector into HEK-293T cells cultured in a 24-well plate. After 48 h of incubation, (D, upper panel) luciferase intensities were measured and normalized to β-galactosidase activities (**p < 0.01), and (D, lower panel) expression levels of p-Akt, Akt, Flag-FOXO1, p-Flag-FOXO1, and HBP1 were measured by Western blotting with α-tubulin as an internal control. (**E**) Effect of different FOXO1 domain on *HBP1* promoter activity. A 2-kb *HBP1* promoter-luciferase construct and a β-galactosidase plasmid were co-transfected with a pcDNA3 empty vector, FOXO1, FOXO1-H215R or constitutively active FOXO1-AAA vector (0.2 μg) into HEK-293T cells. After 48 h of transfection, luciferase intensities were measured and normalized to β-galactosidase activities (*p < 0.05; **p < 0.01).

The EGFR/PI3K/Akt signaling is often up-regulated in malignant oral cancer [21, 22]. Transcriptional activity of FOXO1 can be suppressed through Akt-mediated post-translational modifications [19]. In previous studies, we demonstrated that activation of Akt leads to down-regulation of HBP1 expression (Figure 4A in [15]), and inhibition of Akt phosphorylation by LY294002, a chemical inhibitor of PI3K, results in increased protein level of FOXO1 (Figure 4F in [23]). Hence, we studied whether FOXO1 activation of the *HBP1* promoter is also under the control of the upstream regulator Akt in the HSC-3 oral cancer cell line with an aberrant activation of the EGFR/PI3K pathway [23]. Indeed, administration of LY294002 (20 μM) to HSC-3 cells potently suppressed Akt and FOXO1 phosphorylation with a concomitant increase in HBP1 protein expression (Figure 3A). LY294002 also caused increased mRNA level of HBP1 in HSC-3 cells (Figure 3B). In addition, the reporter gene assay revealed that LY294002 enhanced the FOXO1-mediated *HBP1* promoter activity in a dose-dependent fashion (Figure 3C). Then we tested the direct effect of Akt on FOXO1-mediated HBP1 expression. Overexpression of either wild-type (Akt1) or constitutively active Akt1 (Myr-Akt1) potently abolished FOXO1-induced activation of the 2-kb *HBP1* promoter (upper panel, Figure 3D) and the expression of *HBP1* protein (lower panel, Figure 3D). However, the Akt mutant (Akt1 T308A/S473A) had no significant effect on FOXO1-mediated activation of *HBP1* promoter and protein expression (Figure 3D). These results suggest that Akt phosphorylation of FOXO1 might modulate the transcriptional activity of the *HBP1* promoter. We further employed FOXO1-AAA, a constitutively active FOXO1 mutant, with triple mutation on the Akt phosphorylation sites, T24A, S256A, and S319A, [24] to test this hypothesis. FOXO1-AAA overexpression showed a stronger enhancement effect on the promoter activity of *HBP1* than that of wild-type FOXO1 overexpression in HEK 293T cells (Figure 3E). In addition, the result from the DNA-binding defective FOXO1 (H215R) [24] indicated that this domain is also crucial for the full activation of FOXO1 on the *HBP1* promoter (Figure 3E). These data demonstrated that both the protein activity and its DNA-binding ability of FOXO1 are crucial for the FOXO1-mediated HBP1 activation. In addition, the FOXO1-HBP1 axis is a crucial downstream effector of the EGFR/PI3K/Akt signaling pathway in oral cancer.

**Figure 4:**
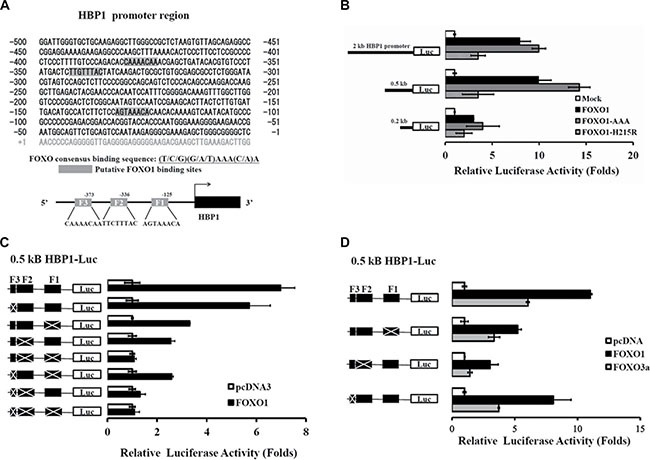
Identification of FOXO1 response elements in the HBP1 promoter (**A**) Schematic diagram of the *HBP1* proximal promoter containing three potential FOXO1 binding sites at positions –132 to –125, –343 to –336, and –380 to –373 bp (depicted as F1, F2, and F3, respectively) from the transcriptional start site as predicted by MAPPER Search Engine. (**B**) Relative activation of FOXO1 and its mutants on various lengths of the native *HBP1* promoters. HEK-293T cells were co-transfected with luciferase reporters fused with the indicated lengths of the *HBP1* promoter as well as the expression plasmid of wild-type FOXO1 or FOXO1 mutant (FOXO1-AAA or FOXO1-H215R). Luciferase activity normalized to β-galactosidase was determined 24 h after transfection and represented as means ± S.E.M. from three separate experiments. (**C**–**D**) A 0.5-kb *HBP1* promoter-luciferase construct with a series deletion combination of F1, F2 and/or F3 was co-transfected with a (C) FOXO1 or (D) FOXO1 or FOXO3a expression plasmid into HEK-293T cells. After 24 h of incubation, luciferase activity relative to control (empty vector) was determined after normalization to β-galactosidase.

### Identification of the FOXO1 response elements in the *HBP1* promoter

Next, we studied if the regulation of FOXO1 in the transcription of HBP1 is through direct binding to the *HBP1* promoter. Three putative FOXO1 binding sites with a core recognition sequence (T/C/G)(G/A/T)AAA(C/A)A or TT(G/A)TTT(G/A)(G/C)[24, 25] were found on the upstream region –132 to –125, –343 to –336, and –380 to –373 bp from the *HBP1* transcription start site (denoted as F1, F2, and F3, respectively) (Figure 4A). To test the effect of these potential FOXO1 binding sites, first, we co-transfected a pGL3-based luciferase reporter gene carrying a 0.2 kb, 0.5 kb, or 2 kb *HBP1* promoter region along with various FOXO1 cDNA clones into HEK-293T cells, and we found that FOXO1 was able to enhance the *HBP1* promoter activity of all three regions with a maximal activation for the 0.5 kb promoter (Figure 4B). This result suggests that FOXO1 binding sites might be located within the proximal region of the *HBP1* promoter. To further differentiate and pinpoint the significance of these three potential FOXO1 sites, we carried out a serial deletion on F1, F2, and/or F3 in the 0.5 kb HBP1-luciferase reporter plasmid as depicted in Figure 4C. Loss of F1, F2, or F3 diminished FOXO1-mediated activation, and the deletion of both F1 and F2 completely abolished FOXO1 activation of the *HBP1* promoter (Figure 4C). Next we examined if FOXO3a also mediated the activity of the *HBP1* promoter through these binding sites. As shown in Figure 4D, F2 box is crucial for FOXO3a-mediated activation of the *HBP1* promoter while both F1 and F2 boxes are important for FOXO1.

**Figure 5:**
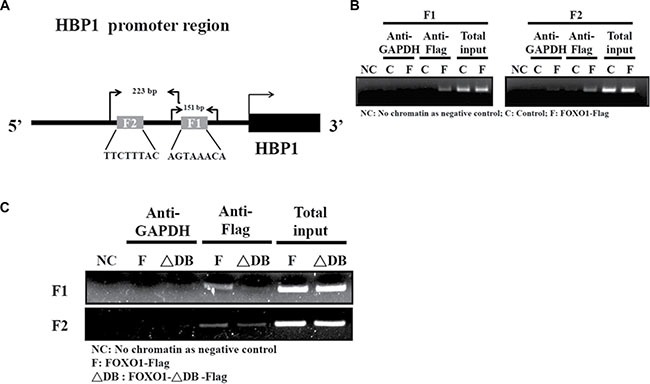
FOXO1 occupies its consensus binding sites in the endogenous HBP1 promoter (**A**) A schematic diagram indicates two primer sets designed for the PCR detection and the expected sizes of the PCR products in the chromatin immunoprecipitation (ChIP) assay. (**B**–**C**) ChIP assay was performed to determine the formation of FOXO1-*HBP1* promoter complex in (B) HEK-293T and (**C**) HSC-3 cells. Cells were transfected with a pcDNA3 (Control; C), pcDNA3-FOXO1-Flag (FOXO1-Flag; F), or pcDNA3-FOXO1-ΔDB-Flag (FOXO1-ΔDB-Flag; ΔDB) plasmid for 48 h, followed by sequential fixation, immunoprecipitation with anti-Flag or GAPDH antibody, and PCR analysis with the primer sets indicated in (A).

Lastly, to investigate whether FOXO1 binds to the human *HBP1* promoter with the context of native human chromatin, chromatin immunoprecipitation (ChIP) assays were performed in HEK-293 cells [24]. Figure 5A depicted the 151 bp and 223 bp primer sets spanning the F1 and F2 boxes in the human *HBP1* promoter starting at –125 bp and –336 bp, respectively, for the PCR analysis. FOXO1-Flag overexpression protein formed a complex with either region of the endogenous human *HBP1* promoter, whereas the *HBP1* promoter binding signal was barely detected in the negative control, GAPDH immunoprecipitation (Figure 5B). In contrast, FOXO1-ΔDB –Flag, lacking the DNA binding domain, obviously diminished the binding of FOXO1 to the human *HBP1* promoter in HSC-3 cells (Figure 5C). These data confirm that FOXO1 binds to the native human *HBP1* promoter *in cellulo*.

### FOXO1-mediated activation of HBP1 expression suppresses tumor cell proliferation and invasion

**Figure 6:**
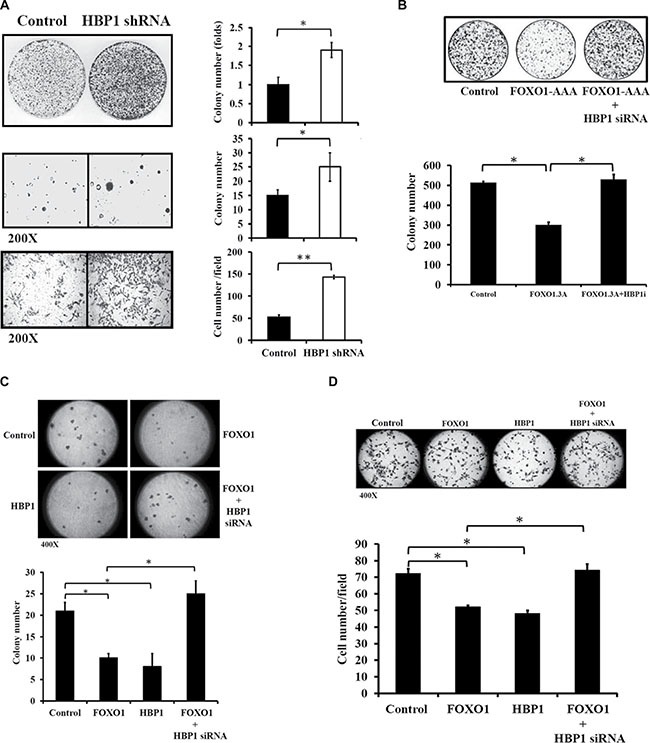
The role of FOXO1-mediated HBP1 expression in oral cancer malignancy (**A**) HBP1 knockdown enhanced colony formation, anchorage-independent growth, and cellular invasion. HSC-3 cells were transfected with either pLKO or pLKO-HBP1 shRNA #76 plasmid (Sigma) in 60 mm culture dishes, followed by assays for colony formation, anchorage-independent growth, and cellular invasiveness. Colony number or invading cells were quantified and *p < 0.05 indicated a significant difference between the two designated groups. (**B**) Effect of the FOXO1-HBP1 axis on colony formation. HSC-3 cells transfected with FOXO1-AAA in the presence or absence of HBP1 siRNA were cultured in growth media for 7 days. Cell colonies were stained with crystal violet and the intensity was quantified at 540 nm (*p < 0.05 as compared with Control). (**C**–**D**) Effect of the FOXO1-HBP1 axis on invasive potential of oral cancer cells. HSC-3 cells (3 × 10^4^) transfected with FOXO1-Flag (FOXO1), HBP1 cDNA (HBP1), or FOXO1-Flag along with either scramble siRNA or HBP1 siRNA (FOXO1 + HBP1 siRNA) were subjected to (C) colony formation in soft agar and (D) invasion assay as described in Materials and Methods. *p < 0.05 indicated a significant difference between the two designated groups.

Next, we examined whether FOXO1-mediated expression of HBP1 modulates tumorigenic growth and metastatic potential in oral cancer. As a downstream effector, HBP1 knockdown potently promoted cell malignancy as demonstrated by increased colony and invading cell numbers in HSC-3 cells (Figure 6A). Ectopic overexpression of constitutively active FOXO1 (FOXO1-AAA) significantly suppressed colony growth in HSC-3 oral cancer cells; however, HBP1 knockdown alleviated the suppressive effect of FOXO1-AAA on colony formation (Figure 6B). To further examine the role of FOXO1-mediated HBP1 expression in metastatic potential, both colony growth in soft agar and Matrigel invasion assay were performed in HSC-3 cells. Overexpression of either FOXO1 or HBP1 significantly decreased the ability of HSC-3 cells to form colonies in soft agar as compared with the vector control, while HBP1-specific siRNA diminished the suppressive effect of FOXO1 (Figure 6C). The data from the Matrigel invasion assay also exhibited the similar pattern (Figure 6D). Taken together, these data support HBP1 as a downstream target of FOXO1, which contributes to FOXO1-mediated inhibition of malignancy in oral cancer.

## DISCUSSION

The PI3K/Akt pathway is commonly altered in human cancer [26]. The constitutive activation of the PI3K/Akt axis is mainly due to either gain-of-function mutations in *PI3KCA*, loss-of-function mutations in *PTEN*, or amplification of *EGFR*. Therefore, a large group of tumors with molecular alterations in the PI3K/Akt pathway is therapeutically targetable with PI3K inhibitors [27]. Growing evidence supports HBP1 as a key negative downstream regulator of the PI3K/Akt pathway. In a previous study, we demonstrated that HBP1 protein expression was down-regulated upon ectopic expression of a constitutive form of Akt in oral cancer cells [15]. In the present study, we further found that the PI3K inhibitor LY294002 significantly up-regulated HBP1 expression with a concomitant decrease in FOXO1 phosphorylation in HSC-3 oral cancer cells (Figure 3A–3B). Similar observation was also reported by Coomans de Brachene and colleagues that the PI3K inhibitor LY294002 or other Akt inhibitor (Akt inhibitor VIII or MK 2206) caused increased mRNA or protein expression of HBP1 in either eosinophilic leukemia or colon carcinoma cells [18]. In addition, HBP1 expression coincided with the FOXO activity under regulation by Akt inhibitor GSK690693 [28]. Concomitant reduction of mRNA expression levels of FOXO1 with HBP1 were also observed in a set of breast tumors [18]. In the current study, we found a decreased FOXO1 mRNA level in invasive oral tumor specimen, and the combination of decreased mRNA expression levels of FOXO1 and HBP1 may predict invasiveness of oral cancer.

Through direct binding to the consensus binding sites in the promoter regions, the FOXO transcription factors regulate the transcription of their target genes [29]. The potential FOXO1 binding sites in the *HBP1* promoter were found –132 to –125, –343 to –336, and –380 to –373 bp upstream of the *HBP1* transcription start site (denoted as F1, F2, and F3, respectively) (Figure 4A) using the MAPPER Search Engine (http://genome.ufl.edu/mapper/). Employing reporter genes carrying different regions of the *HBP1* promoter, we found that a 0.5 kb *HBP1* promoter, containing all three possible FOXO1 sites, displayed the strongest FOXO1 activation; however, a 0.2 kb promoter, which contains only one potential FOXO1 binding site, F1, was able to be stimulated by FOXO1, suggesting that this site is also a functional FOXO1 regulatory element (Figure 4B). Next we tested a series of single and combined deletions in the 0.5 kb *HBP1* promoter, and our results indicated that F1 and F2 are the major FOXO1 sites, while F3 is necessary for the full FOXO1 activation of the *HBP1* promoter (Figure 4C). Interestingly, FOXO1-H215R, a FOXO1 mutant defect in the DNA binding domain, was able to activate the *HBP1* promoter although the induction was much weaker than that of the wild-type FOXO1 (Figure 4B), suggesting that HBP1 transcription can be regulated by FOXO1 through either direct or indirect binding. Indeed, FOXO1 can interact with other transcription factors to transcriptionally repress cyclin D1 (*CCND1*) and D2 (*CCND2*) [30] or activate cyclin G2 (*CCNG2*) and p130 (*Rbl2*) [31].

The *HBP1* promoter seems to respond preferentially to FOXO1 than FOXO3a. To differentiate the role of FOXO1 and FOXO3a in the activation of the *HBP1* promoter, a series of reporter gene assays were employed. The results revealed that FOXO1 is a stronger activator than FOXO3a for either 2-kb or 0.5-kb *HBP1* promoter (Figures 2E, 4D). Of special note, although both FOXO1 and FOXO3a are able to induce the *HBP1* promote activity, our data further indicate that the consensus binding site F2 is absolutely a prerequisite for the FOXO3a action on the *HBP1* promoter (Figure 4D), which is in an agreement with the finding by Coomans de Brachene et al. [18]. However, in the absence of F2, the binding site F1 still contributed to at least 2-fold of the FOXO1 activation on the *HBP1* promoter (Figure 4B–4D). Taken together, our data support FOXO1 as a crucial upstream regulator of the *HBP1* gene transcription. Future study may reveal if the FOXO1-HBP1 axis and the FOXO3a-HBP1 axis share function redundancy, or one may be more active than the other under certain biological context.

Both FOXO1 and HBP1 are potential tumor suppressor genes; decreased FOXO1 expression [3] or loss-of-function *HBP1* mutation [10, 11] is often associated with tumorigenesis. FOXO1 and HBP1 may exert their tumor suppression function through the induction of growth arrest and apoptosis [4–6, 12, 14, 15, 23]. Here, we showed that HBP1 can function as a downstream effector of FOXO1-mediated growth inhibition in oral cancer; HBP1 knockdown alleviated the suppressive effect of FOXO1 on colony formation (Figure 6B–6C). In addition, accumulated evidence also supports a role of FOXO1 and HBP1 in metastatic potential of cancer cells. Negative regulation of FOXO1 in cellular migration or invasion has been reported in various types of cancer, including prostate [32], and breast cancer [33]. Similarly, HBP1 knockdown hastens cell migration and invasion in breast and prostate cancer cells [11, 14]. In the current study, we further provide evidence that the FOXO1-HBP1 axis may suppress the invasiveness of oral cancer cells (Figure 6D). Take together, the FOXO1-HBP1 axis appears to be a potential therapeutic target of anticancer reagents in oral cancer.

## MATERIALS AND METHODS

### Reagents and antibodies

All chemicals were purchased from Sigma (St. Louis, MO) and antibodies were from Santa Cruz Biotechnology (Santa Cruz, CA), respectively, unless specified otherwise. Antibody for HBP1 was from Novus Biologicals (Littleton, CO), and α-tubulin was from Abcam (Cambridge, MA). PVDF membranes and ECL detection reagents for Western blotting analysis were purchased from Perkin Elmer Life Sciences, Inc. (Waltham, MA). Dual-light^®^ system was from Applied Biosystems (Foster City, CA). PI3K inhibitor LY 294002 was purchased from Sigma (St. Louis, MO). Small interfering RNA (siRNA) molecules specific for HBP1 was purchased from Invitrogen (Carlsbad, CA).

### Plasmids

Expressing plasmids, including pcDNA3-Flag-FKHR(FOXO1)-Delta DB, pcDNA3-flag FKHRL1 (FOXO3a), pcDNA3-Flag-HA, pcDNA3-Flag-HA-Akt1, pcDNA3-Myr-HA-Akt1, and pcDNA3-T7-Akt1-T308A-S473A [34] were purchased from Addgene (Cambridge, MA, USA). The HBP1 expressing plasmid pEF-BOS-HA-HBP1, and FOXO1 expressing plasmids pcDNA3-A3-FOXO1-Flag pcDNA3-WT-FOXO1-Flag and pcDNA3-H215R-FOXO1-Flag were kindly provided by Dr. Amy S. Yee and Dr. Brian Schaffhausen (Tufts University, USA), respectively.

### Cell culture and treatment

For human oral squamous carcinoma cell lines, HSC-3 and TW206 were kind gifts of Dr. Hsin-Ling Yang and SAS and OECM-1 cells were kindly provided by Dr. Jang-Chang Lee at China Medical University (Taichung, Taiwan). HOK human oral keratinocytes and HEK (Homo sapiens embryonic kidney)-293T cells were obtained from Drs. Shieh Tzong-Ming and Tzong-Der Way at China Medical University, respectively (Taichung, Taiwan). HSC-3 and OECM-1 cells were maintained in Dulbecco's Modified Eagle Medium (DMEM)/F-12 and RPMI 1640 medium, and SAS, and 293T cells were maintained in DMEM, respectively, supplemented with 10% fetal bovine serum (FBS) and 1% antibiotic-antimycotic. Cells were cultured in a 37^°^C, 5% CO_2_ incubator. All cell culture reagents were from Invitrogen (Carlsbad, CA), unless indicated otherwise.

### Colony formation assay

HSC-3 cells after transfection were cultured in growth media in 60 mm dishes for 7 days, and then washed with PBS, fixed with 10% formalin (Mallinckrodt Chemicals, St. Louis, MO) for 10 min, and stained with 0.05% crystal violet (Panreac Quimica S.A.U.) for 30 min. Dishes were scanned for colony counting and crystal violet stain was dissolved in 100% methanol for optical density measurement at 540 nm.

### Reporter assay

The pGL3-luc plasmids containing 2037 (2 kb), or 526 (0.5 kb), or 166 (0.2 kb) bp *HBP1* promoter and RSV-β-galactosidase plasmids were kindly provided by Dr. Amy S. Yee, Tufts University, USA. HEK-293T cells (3 × 10^4^ cells) cultured in a 24-well plate were transfected with pDL3-HBP1-luc (0.4 μg) and RSV-β- galactosidase (0.1 μg) along with indicated vectors for 24 h and then cell lysates were collected in lysis solution (Applied Biosystems, Carlsbad, CA) according to the manufacturer's protocol. The Dual Light^®^ System (Applied Biosystems) was used to quantify luciferase and β-galactosidase activities.

### Tissue sample preparation

As previously described, [35] fresh oral tumor specimens were collected from oral cancer patients and stored in liquid nitrogen. Tumor tissues containing > 85% tumor cells based on the staining results were qualified for further RNA extractions. Acquisition of tissue specimens was approved by the institutional review board of the China Medical University Hospital. A total of 30 oral tumor specimens was obtained from the tissue bank at China Medical University Hospital (Taichung, Taiwan). Total RNA extracted from 9 normal oral epithelial counterparts was used as reference samples. All 30 tumor specimens were divided into two groups, non-invasive (*n* = 18, p*N* = 0, no metastasis) and invasive (*n* = 12, pN > 0, lymph node metastasis).

### Reverse transcription-polymerase chain reaction, real-time PCR, and PCR

Total RNA was extracted using RNeasy^®^ Mini kit (Qiagen) according to the manufacturer's instructions. RT-PCR was performed using SuperScript™ III One-Step RT-PCR System with Platinum^®^ Taq DNA Polymerase Kit (Invitrogen). The following primers were used: human *HBP1*, 5′-ATCATCTCCTGTACACATCATAGC-3′(F) and 5′-CATAGAAAGGGTGGTCCAGCTTAC-3′(R); *18S*, 5′-GTCTGTGATGCCCTTAGATG-3′(F) and 5′-AGCT TATGACCCGCACTTAC-3′(R). Primer sequences for real-time PCR analysis of oral tumor specimens were: *HBP1*, 5′-GAACCAATTCAGGCTCACA-3′(F) and 5′-TC AAGACTCAATGCTATCAGTATC-3′(R); *FOXO1*, 5′-AA GAGCGTGCCCTACTTCAA-3′(F) and 5′-CTGTTGTT GTCCATGGATGC-3′(R). Primer sequences for PCR analysis in ChIP assays were: *HBP1*-125, 5′-TCTTTCG CCCTCTTATTGA-3′ (F) and 5′-GAACTGCCATTCGG TTCTTC-3′ (R); *HBP1*-336, F 5′-TTGTCCCAGACAC CAAAACA -3′(F) and 5′-GGATTGGACTATTGCCG AGA-3′(R).

### Matrigel invasion assay [36]

Matrigel inserts for a 24-well chamber were purchased from BD Biosciences (Bedford, MA) and operated according to the manufacturer's protocol. HSC-3 cell suspensions (3 × 10^4^ cells) after transfection with indicated plasmids were seeded onto the upper chamber in a serum-free medium and 10% FBS-containing medium was added to the lower chamber to serve as a chemoattractant. After 24 h of incubation in a 37^°^C, 5% CO_2_ incubator, the non-invading cells from the upper chamber were removed using cotton swabs while the invading cells on the lower surface were fixed with 100% methanol, stained (Giesma in 20% ethanol), and counted. The invading cells were photographed and counted in five, randomly selected microscopic fields (200X magnification). Cell invasion was photographed under 400× magnification. Error bars in Figure 6C represent the variation of the cell numbers between the selected fields.

### Colony formation in soft agar [14]

HSC-3 cells (5 × 10^3^ cells) after transfection with indicated constructs were suspended in 0.35% agar (DNA grade agarose, BIOMAN Scientific Co., Ltd, Taiwan) in DMEM containing 10% FBS and plated in 35 mm Petri dishes with a 0.5% agar bottom layer. After 14 days of incubation at 37^°^C in a humidified incubator, the colonies > 200 μm in diameter were counted microscopically within the field of a X40 objective lens (Olympus 1 × 71). Each bar represents the mean number of colonies (×40 field) ± S.E.M. from three independent experiments.

### Chromatin immunoprecipitation (ChIP) assays

Cells transfected with empty vector or vectors encoding FOXO1-Flag or FOXO1-Delta DB were subjected to chromatin immunoprecipitation experiments performed with the Chromatin Immunoprecipitation (ChIP) Assay Kit (Millipore), following the manufacturer's instructions. Anti-Flag and control anti-GAPDH antibody were purchased from Santa Cruz Biotechnology (Santa Cruz, CA) and DNA was extracted using DNA extraction kit (Favorgen biotech corp., Taiwan). PCR reactions were conducted using the Fermentas PCR kit (Thermo scientific, PA, USA), following the cycling conditions: 1 cycle (95°C 2 min), 30 cycles (95°C 30 s, 58°C 30 s, 72°C 1 min), and 1 cycle (72°C 5 min) with the indicated primers. Migration of PCR products was performed in 2% agarose gels.

### Statistical analysis

Data expressed as mean ± SD or mean ± SEM were calculated from at least three independent experiments. Statistical significance was analyzed using Student's *t* test. Results were considered significantly different at p < 0.05.

## SUPPLEMENTARY MATERIALS FIGURES


