# Supplementary material for: Transcription factor HBP1 is a direct anti-cancer target of transcription factor FOXO1 in invasive oral cancer
Source: Oncotarget. 2017 Jan 14;8(9):14537–48. doi: 10.18632/oncotarget.14653 (PMC5362424; doi:10.18632/oncotarget.14653)
Supplement: Supplementary file 1 [file oncotarget-08-14537-s001.pdf]

Transcription factor HBP1 is a direct anti-cancer target of transcription factor FOXO1 in invasive oral cancer

Supplementary Materials

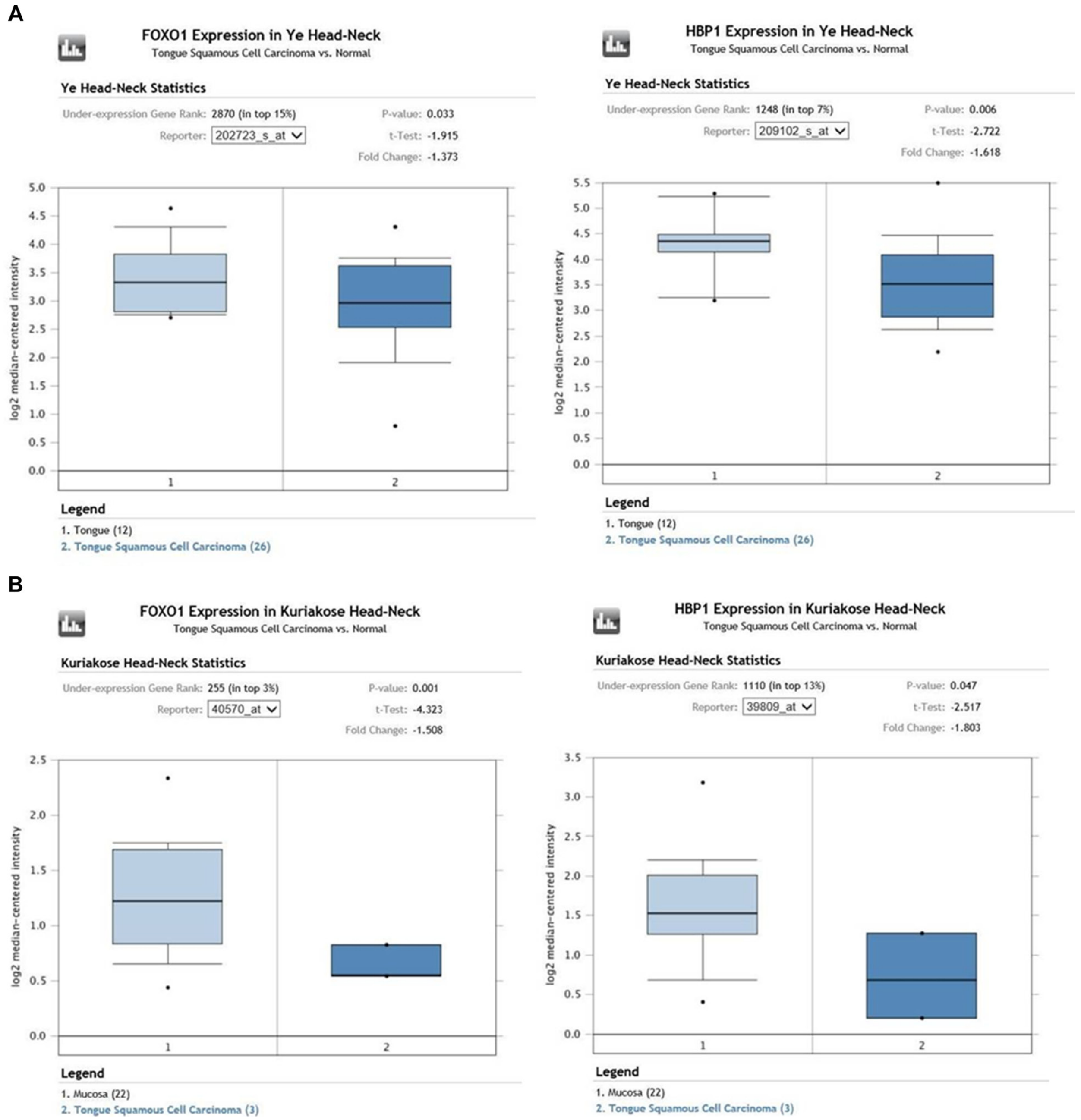

Supplementary Figure 1: Down-regulation of both FOXO1 and HBP1 expression levels in human tongue squamous cell carcinomas on the Oncomine database. Both FOXO1 and HBP1 mRNA levels in human tongue squamous cell carcinomas and normal tissues from (**A**) Ye and (**B**) Kuriakose head-neck dataset were plotted. The boxes represent the 25th through 75th percentiles and the horizontal lines inside the boxes represent the medians. The whiskers represent the 10th and 90th percentiles, and the asterisks represent the end of the ranges. The Student *t* test was conducted using the Oncomine software.
